# Single-particle mass spectrometry with arrays of frequency-addressed nanomechanical resonators

**DOI:** 10.1038/s41467-018-05783-4

**Published:** 2018-08-16

**Authors:** Eric Sage, Marc Sansa, Shawn Fostner, Martial Defoort, Marc Gély, Akshay K. Naik, Robert Morel, Laurent Duraffourg, Michael L. Roukes, Thomas Alava, Guillaume Jourdan, Eric Colinet, Christophe Masselon, Ariel Brenac, Sébastien Hentz

**Affiliations:** 1 Univ. Grenoble Alpes, CEA, LETI, 38000 Grenoble, France; 2 Centre for Nano Science and Engineering, Indian Institute of Science, Bangalore, 560012 India; 3 Univ. Grenoble Alpes, CEA, CNRS, Grenoble INP, INAC-Spintec, 38000 Grenoble, France; 4 Kavli Nanoscience Institute and Departments of Physics, Applied Physics, and Bioengineering, California Institute of Technology, MC 149-33, Pasadena, CA 91125 USA; 5 Université Grenoble-Alpes, 38000 Grenoble, France; 6 CEA, BIG, Biologie à Grande Echelle, 38054 Grenoble, France; 7 Inserm, Unité 1038, 38054 Grenoble, France; 8 Present Address: APIX Analytics, 7 parvis Louis Néel – CS20050, 38040 Grenoble, France

## Abstract

One of the main challenges to overcome to perform nanomechanical mass spectrometry analysis in a practical time frame stems from the size mismatch between the analyte beam and the small nanomechanical detector area. We report here the demonstration of mass spectrometry with arrays of 20 multiplexed nanomechanical resonators; each resonator is designed with a distinct resonance frequency which becomes its individual address. Mass spectra of metallic aggregates in the MDa range are acquired with more than one order of magnitude improvement in analysis time compared to individual resonators. A 20 NEMS array is probed in 150 ms with the same mass limit of detection as a single resonator. Spectra acquired with a conventional time-of-flight mass spectrometer in the same system show excellent agreement. We also demonstrate how mass spectrometry imaging at the single-particle level becomes possible by mapping a 4-cm-particle beam in the MDa range and above.

## Abstract (web summary)

Nano-electro-mechanical system-based mass spectrometry holds promise for detecting supramolecular assemblies at large molecular weights, but its efficiency is too poor to be practical. Sage et al. overcome this problem using a nanomechanical resonator array, which significantly decreases detection time.

## Introduction

Mass spectrometry (MS) has been one of the fastest-growing analytical techniques over the past two decades^1,2^ and has become an essential tool in a broad variety of fields^3–5^. MS is particularly well suited to the analysis of light molecules: it is based on ionization, which raises issues for high-mass species^6^. Routine use of MS in the MDa (~1.66 ag, 1 ag = 10^−21^kg) to GDa (~1.66 fg, 1 fg = 10^−18^kg) range remains challenging: while currently out of reach for commercial instruments, a few specialized systems have shown the ability to study supramolecular assemblies in the one to tens of MDa mass range^7,8^. In this mass range, interesting results have been recently obtained with unconventional MS architectures like charge detection systems^9,10^ also based on ionization of species. In parallel, mass sensing using nanomechanical resonators has been performed for the last fifteen years with a variety of devices^11^ and a mass limit of detection of a few Daltons has been reported using a carbon nanotube^12^. Nano-electro-mechanical systems (NEMS) operate best in the MDa to GDa range, and real-time acquisition of NEMS-MS for single proteins has been demonstrated with top-down silicon resonators^13^. The effect of particle stiffness on the resonator’s response has been shown recently^14^. Moreover, an important milestone has been reached with the demonstration of MS of particles regardless of their charge with NEMS^6^, which can circumvent issues associated with ionization of species, in particular transfer efficiency. The excellent mass limit of detection obtained with nanomechanical devices comes at the cost of an extremely reduced capture cross-section, and this is of course all the more true for bottom-up devices like carbon nanotubes. One of the main challenges to overcome to perform NEMS-MS analysis in a practical time frame stems from the size mismatch between the analyte beam and the nanomechanical detector area^13,14^. It is therefore of crucial importance to significantly increase the capture cross-section of resonators while maintaining their outstanding performance. Capturing a larger proportion of particles will also decrease the amount of sample required to perform an analysis, which can be a requirement for some biological species. In the past, gas sensing was demonstrated with dense and large arrays of identical interconnected NEMS^15^. In this case, gas molecules adsorb homogeneously onto the surface of all NEMS within the array, which operate collectively and simultaneously. This is not suitable for NEMS-MS, as information about each single device is lost in the collective operation of the array: a single particle would shift the frequency of only one device, and this information would be averaged over the whole array.

We report here the demonstration of NEMS-MS with arrays of individually addressed nanomechanical resonators where the number of inputs/outputs for the whole array is the same as that of a single resonator. While all resonators within an array are interconnected via two metal levels, each resonator is designed with a distinct resonance frequency which becomes its individual address. NEMS within an array operate in multi-mode^6^ and retain the same mass resolution as a single resonator. Using such an array, mass spectra of metallic aggregates have been acquired with excellent speed due to a significantly enhanced capture cross-section compared to individual resonators. Spectra acquired with a conventional time-of-flight (TOF) mass spectrometer in the same system show excellent agreement. As individual information for each resonator within the array is retained, we demonstrate spatial imaging of a particle beam at the single-particle level.

## Results

### Frequency addressing of nanomechanical resonators

**Fig. 1:**
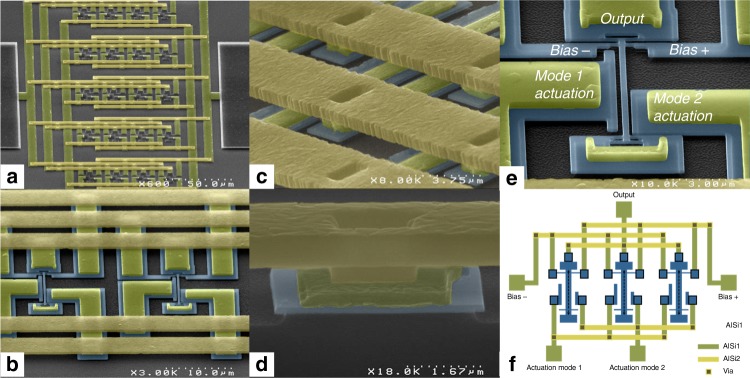
Array of nanomechanical resonators. SEM images of 5×4 NEMS array used for nanomechanical mass spectrometry. Typical horizontal and vertical pitches are 20 and 55 µm respectively. **a** General view of the array, **b** zoom on two resonators (silicon is false-colored in deep blue), and their metal interconnects (AlSi). **c**, **d** zoom-in on interconnects and via. The first metal level is colored in deep yellow, the second one in light yellow. **e** Typical doubly clamped in-plane resonator used in this study. The beams are designed to resonate around 30 MHz for mode 1 and 80 MHz for mode 2. Typical dimensions for the resonant beam are: 160 nm (thickness), 300 nm (width), and 5–10 µm (length). In-plane motion transduction is performed using piezoresistive nanogauges in a bridge configuration to allow background cancellation. Electrodes are specifically patterned for efficient mode 1 and mode 2 actuation. For a resonance frequency *f*_0_, bias voltages at *f*_0_+∆*f* are applied to both nanogauges (with 180° dephasing). Tension/compression in the gauges mix their resistance change to obtain a downmixed differential output voltage at ∆*f*, typically around a few 10’s of kHz. **f** Schematic of the interconnect layout. Each resonator has a unique beam length, hence a unique resonance frequency

We use monocrystalline silicon resonators fabricated from silicon-on-insulator wafers with very large scale integration processes^16^. The resonators are electrostatically actuated and use a differential piezoresistive readout. Particles landing on a resonator add to its total mass and cause its resonance frequency to down-shift. As these frequency shifts also depend on the landing position on the resonator’s surface, the frequencies of two resonance modes are monitored simultaneously to resolve the two unknowns (i.e., mass and position) in real time^6^. Two asymmetric drive electrodes are used to simultaneously operate each resonator on its two first resonance modes. Every input/output of all resonators within an array is interconnected across resonators: for instance, the output pad of the array is electrically connected to the output pads of all resonators within the array. With five inputs/outputs per resonator, this is only made possible by using two metal levels and vertical interconnects (Fig. 1a–d). The number of electrical pads for the whole array is thus the same as that for a single resonator (Fig. 1f). One obvious advantage of such a configuration is that the same measurement setup can be used for both single resonators and arrays (provided a sufficiently high measurement bandwidth), without the need for complex wire-bonding or addressing (see fabrication details in “Array fabrication” in the Methods section).

Retrieving individual information corresponding to each resonator within the array as required by NEMS-MS is performed by “frequency addressing”: distinct resonance frequencies for each resonator are obtained by slightly varying their length. The frequency pitch needs to be large enough so that resonance peaks do not overlap after fabrication due to process uncertainty as well as after mass deposition which results in downshifts in resonance frequency. The choice of pitch is a trade-off between risk of spectrum overlap, number of resonators within the array and measurement bandwidth. Arrays of 20 (5×4) resonators are fabricated with typical spatial pitches of 20 µm and 55 µm in the horizontal and vertical direction, respectively. Resonance frequencies of our arrays typically range from 20 to 45 MHz for mode 1, and 70 to 120 MHz for mode 2 with frequency pitches in the 500 kHz range (length difference in the 150 nm range).

**Fig. 2:**
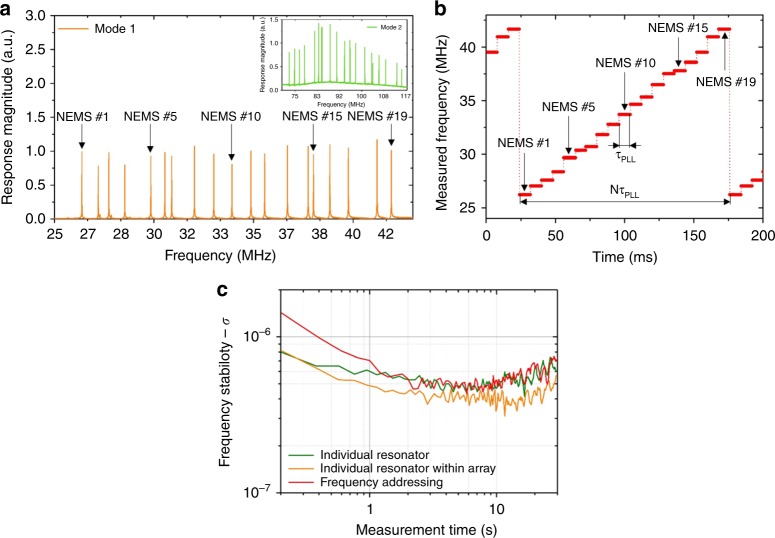
Frequency-addressing technique. **a** An open loop response of an array of 20 NEMS is recorded for mode 1 and mode 2 (inset). Each peak corresponds to the resonance of a single NEMS resonator for which resonance frequency and phase reference can be used as an address. We are showing here an example with only 19 resonance peaks: one resonator in the array failed after a long period of operation, as confirmed by scanning electron microscopy (SEM) observation. Yet, the array as a whole could still be operated without performance degradation, demonstrating the robustness of the parallel architecture. **b** The resonance frequency of every single resonator in the array is sequentially monitored over time: a PLL locks onto a given resonator, registers its current resonance frequency after a given idling time *τ*_PLL_ (here 8 ms) and then switches to the next resonator. The duty cycle of a whole array is then *Nτ*_PLL_ (here 152 ms with *N* = 19 NEMS). From the recorded data points, individual frequency time traces can be extracted, and their frequency stability calculated. **c** Frequency stabilities obtained using a single individual resonator (not in array, green), a resonator of strictly identical dimensions within an array without frequency addressing (yellow) and the same resonator with frequency addressing (red). See “Frequency stability measurements” in the “Methods” for details on the selected frequency stability estimator. The three plots appeared identical within measurement uncertainty: the parallel architecture of our arrays along with the frequency-addressing technique allows reaching the regime where frequency fluctuations set the frequency stability limit of our resonators, down to similar values as single resonators^17^

An open loop frequency sweep response of the array in vacuum (~10^−5^ Torr) and at liquid nitrogen temperature is performed with a down-mixing scheme (Fig. 2a; Supplementary Figure 1). Resonance peaks are found to be well separated from each other with excellent Signal-to-Background ratios (~55 dB), around 180° phase shifts and quality factors (*Q*) very similar to typical *Q*s of individual resonators^6^ (e.g., 8500 and 7500 in average for the first and second mode, respectively). This is despite the fact that for a given drive voltage, the output signal of the array at a given beam’s resonance frequency scales like $\frac{1}{{N - 1}}$, *N* being the number of resonators within the array (Supplementary Methods). While the frequency pitch is designed to be constant, a spread is observed due to fabrication uncertainties.

Our arrays are operated in closed loop to monitor the resonance frequencies of all resonators sequentially over time (Fig. 2b): initial frequencies and phase references are recorded for every NEMS. A phase lock loop (PLL) subsequently locked onto a given resonator and registered a frequency data point after a given idling time *τ*_PLL_, before switching to the next resonator. The duty cycle of a whole array is therefore *Nτ*_PLL_ where *N* is the number of probed NEMS. Each data point is subsequently assigned to its corresponding resonator and individual time frequency traces could be deduced. Mass determination of a single particle requires the use of the second resonance mode^6^. The same procedure can be simultaneously performed with a second measurement channel (Supplementary Figure 1) and an additional PLL. Both first and second mode frequencies can be thus simultaneously monitored in real time and our arrays of nanomechanical resonators can be used to perform NEMS-MS.

### Frequency stability of the arrays of nanomechanical resonators

Frequency stability is a key parameter to the performance of nanoresonators for mass sensing. In a regime where additive white noise is dominant, based on the simple dynamic range equation, the frequency stability can be expressed in the voltage domain as^17^:

1 $$\frac{{\delta f}}{{f_0}} \cong \frac{1}{{2Q}}\frac{{S_{n}}}{{V_{{\mathrm{out}}}}}\sqrt {{\rm{BW}}}$$

where *Q* is the resonator’s quality factor, *V*_out_ the output signal amplitude at any given NEMS resonance frequency (in *V*), *S*_*n*_ the noise spectral density at the output (in *V* Hz^−1/2^), and BW the measurement bandwidth (in Hz). As mentioned above, the output signal of an individual resonator in array configuration scales like the inverse of the number of devices, $V_{{\mathrm{out}}} \propto \frac{1}{{(N - 1)}}$. In our case, *S*_*n*_ is the sum of lock-in input noise (constant, typically less than 10 nV Hz^−1/2^), of Johnson noise due to the piezoresistive gauges and of thermomechanical noise. All piezoresistances being connected in parallel, Johnson noise scales like $\frac{1}{{\sqrt N }}$. Since Johnson noise for a single device is typically in the same order of magnitude as the lock-in input noise, it becomes negligible for an array. Thermomechanical noise for a single resonator is typically in the same order as Johnson noise or the lock-in input noise. Like output signal, it scales in the voltage domain like $\frac{1}{{(N - 1)}}$and becomes negligible for an array. Finally *S*_*n*_ is dominated by the lock-in input noise, which does not scale with the number of resonators in the array, while the output voltage *V*_out_ is inversely proportional to the latter. In a regime where additive white noise is dominant, the frequency stability of resonators within our arrays is expected to degrade proportionally with the number of resonators $\left( {\left\langle {\frac{{\delta f}}{{f_0}}} \right\rangle \propto (N - 1)} \right)$.

From the individual time frequency traces measured with our sequential closed-loop scheme, the frequency stability of every resonator within the array can be plotted. Figure 2c compares three different cases: the first is the frequency stability of a single resonator (not in an array) with a usual down-mixing scheme. The second is the frequency stability of a resonator of identical dimensions within an array, but operated with the same readout scheme (no frequency addressing). Finally, the third trace corresponds to the same resonator within an array with frequency addressing. The three plots are similar within measurement uncertainty. Yet, in the case of additive white noise, we could expect a factor 20 between the two first cases. We attribute this discrepancy to the presence of resonance frequency fluctuations in the mechanical domain: we have recently shown that the frequency stability of similar silicon single resonators is limited by frequency fluctuations rather than additive white noise^17^, more than two orders of magnitude above what is expected from Eq. (1). The physical origin of these frequency fluctuations is still under investigation: we have discarded instrumentation noise, temperature fluctuations, charge fluctuations, non-linearities, adsorption–desorption noise, molecule diffusion, as well as defect motion in the crystalline lattice. Several mechanisms remain to be studied, among which surface effects. In this regime, the frequency stability does not depend on signal level and depends very weakly on integration time. We find the same behavior with our arrays and using the frequency-addressing technique. It should also be noted that frequency stabilities of all resonators within the array are of very similar levels (Supplementary Figure 4). Finally, the frequency-addressing technique does not degrade the frequency stability of our nanoresonators and the limits of detection in mass of single and arrayed resonators are similar.

### Single-particle mass spectrometry with arrays of nanomechanical resonators

**Fig. 3:**
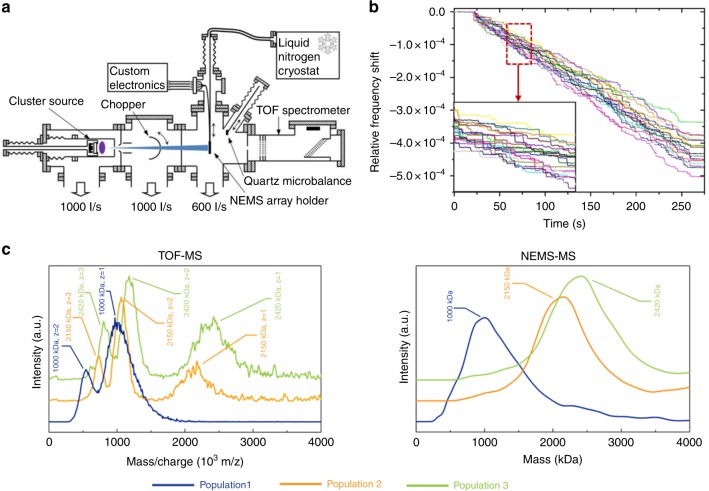
Single-particle Mass Spectrometry with arrays of nanoresonators. **a** Schematic of the setup showing from left to right: the cluster source, an intermediate chamber containing a chopper, the deposition chamber and an in-line TOF mass spectrometer. Both NEMS holder and QCM were retractable, allowing for sequential NEMS-MS, TOF-MS, and QCM measurements with the same operating conditions. **b** Mode 1 relative frequency time traces of an array of 19 NEMS exposed to a flux of tantalum nanoclusters with a mean diameter of 7.2 nm. Inset: zoom-in with frequency jumps induced by single-particle deposition. **c** Comparison of TOF and NEMS-MS with an array of 19 resonators performed with three distinct populations of nanoclusters with mean diameters of 5.8 nm (~1000 kDa), 7.4 nm (~2150 kDa), and 7.7 nm (~2420 kDa), respectively

We subsequently perform single-particle mass spectrometry with our arrays of nanomechanical resonators in a custom setup described in detail elsewhere^6,18^. The system consists of four main vacuum chambers (Fig. 3a): a metallic nanocluster source, an intermediate chamber, a deposition chamber and an in-line TOF mass spectrometer. Metallic nanoclusters are generated using a sputtering gas-aggregation technique with tunable size and deposition rate. Nanoclusters are then expelled into the vacuum deposition chamber (10^−5^ Torr) through a differential pumping stage. The deposition rate is measured using a quartz crystal microbalance (QCM) placed on a translational stage. Upon retraction of this stage, the array of resonators is exposed to the cluster flux. When both NEMS and QCM are retracted, the particle flux enter the acceleration region of the in-line TOF mass spectrometer, where the mass-to-charge distribution of charged particles is measured. The configuration of the deposition chamber allows QCM, TOF-MS, and NEMS-MS measurements sequentially on the same cluster population.

As previously described^6^, we selected tantalum as the analyte as it is both dense (16.6 g cm^−3^) and readily condenses into large clusters. The TOF and NEMS-MS mass spectra acquired for various populations are compared. An example of frequency traces in simultaneous two-mode operation acquired using the frequency-addressing technique during the exposition to cluster beam is shown Fig. 3b. Frequency jumps larger than the frequency stability 3σ are considered as actual particle landing events and converted into a mass probability distribution^6^ (the stiffness of the particles is neglected due to their very small size, the in-plane motion of the resonator and its width-to-thickness ratio^14^). The mass sensitivity of each NEMS is measured by comparing its frequency response to uniform mass deposition with that of a QCM as detailed elsewhere^6^. This is performed for the 19 resonators simultaneously with the frequency-addressing technique (Supplementary Figure 5). Extracted mass sensitivities range from 15.3 to 32.5 Hz/ag for mode 1 and 42.9 to 87.8 Hz/ag for mode 2, which is consistent with the range of the resonator lengths. Monitoring the first two modes of all NEMS is achieved with a PLL response time of 8 ms, yielding a total array response time of 152 ms. Tuning of the nanocluster source parameters and use of a mechanical chopper (Fig. 3a) yield particle adsorption event rates per resonator in the order of one event every few seconds, making the landing of several particles within the duty cycle very unlikely. The mass probability distributions obtained for each event are added for each resonator to build individual mass spectra; the same operation can be performed for all resonators to build the overall array mass spectrum. Mass spectra of three different nanocluster populations acquired by both NEMS-MS and TOF-MS technique are displayed in Fig. 3c. Just like individual nanomechanical resonators^6^, NEMS-MS performed with arrays directly provide the cluster mass distribution independently of the particles charge state. Conversely, TOF-MS provide mass-to-charge ratio distributions corresponding to multiple charge states of the measured clusters, making spectra interpretation less straightforward. Each NEMS-MS spectrum is acquired in only 4 min and yielded ~1000 events. Each resonator detect a similar number of events during this amount of time (~50 events per resonator), demonstrating the 19-fold improvement in capture efficiency due to the use of the array. The overall spectrum provide an accurate mean mass of the cluster populations over a large mass range (530–2400 kDa), with a broader distribution than the TOF spectrometer. As a matter of fact, these experiments are performed in a mass range compatible with operation of the TOF mass spectrometer, i.e., just above the resonator’s mass limit of detection. Over a few MDa, ions are not sufficiently accelerated in order for the ion detector to provide a signal and the TOF spectrometer becomes unable to perform a correct analysis (Supplementary Figure 6). Conversely, the NEMS limit of detection remaining constant with mass, its resolving power (ratio of analyzed mass to mass resolution) improves with increasing mass (Supplementary Figure 7). For a given cluster population, however, arrays yield slightly broader peaks than those of a single resonator. We attribute this to the heterogeneity in both mass sensitivities and mass resolutions of individual NEMS across the array (Supplementary Figures 8 and 9). This effect will become negligible at masses far from this limit of detection, where mass resolution will become negligible compared to measured mass. Nonetheless, our results demonstrate that such frequency-addressed arrays multiply the capture efficiency by the number of individual resonators in the array, in our case, by more than an order of magnitude.

### NEMS mass spectrometry imaging at the single-particle level

**Fig. 4:**
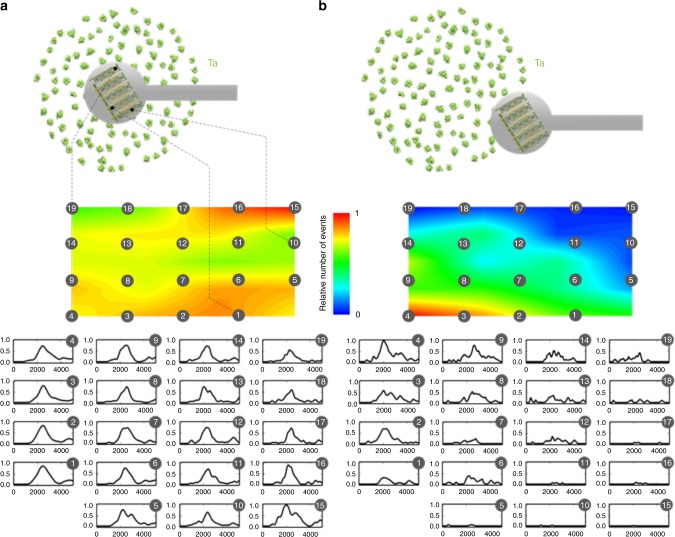
NEMS-MS beam imaging. The NEMS array is placed at the center of the particle beam (**a**) or at the edge (**b**). The event number is measured for each NEMS and plotted on interpolated surface maps for each case. Mass spectra obtained with each individual resonator for a 4 min acquisition are shown. A mechanical chopper is used to adapt the particle adsorption event rate to the array response time. The spectra are displayed as a matrix pattern reproducing the device physical layout (5×4). Each individual plot shows the intensity (a.u.) versus mass (kDa)

The frequency-addressing scheme also provide access to individual information of each resonator. This can be put to use, for example, to obtain a spatial mapping of the particle beam. For this purpose, the 100 µm×250 µm NEMS array is moved to scan the 4-cm-diameter particle beam. Figure 4 shows maps of event number within the array in a given measurement time (here, 4 min), as well as individual spectra obtained with each resonator. These results are presented for two different array locations within the particle beam: close to its center, where the event rate is very homogeneous throughout the array and at the edge of the beam, where there is a clear asymmetry between resonators situated well within the particle beam and the resonators outside of it. The cluster source in our system displays slow drift and day to day variability. As a consequence, we could perform mass analysis at only two different positions in the beam within the time frame available for a stable enough cluster source and for a reasonable number of events on each resonator.

## Discussion

In conclusion, we demonstrate here single-particle nanomechanical mass spectrometry with arrays of NEMS operated with a frequency-addressing scheme. These arrays can comprise several tens of nanoresonators, increasing the total capture cross-section by the same factor. Detection efficiency is today the main limitation of NEMS-based mass spectrometry^14^ with analysis time up to several hours^13^. This time can be reduced by more than an order of magnitude with the frequency-addressing technique, while keeping the same mass limit of detection: frequency stability of the silicon nanoresonators used here being limited by frequency fluctuations, it is not degraded by the frequency-addressing technique. The number of resonators in the array could be further increased, with the caveat that both total input impedance of the array and output voltage would decrease accordingly. Additive white noise would eventually dominate and the frequency stability would degrade. We estimate that this may not be the case for arrays including up to between 50 to 100 nanoresonators. The main price to pay for the frequency-addressing technique is an increase in duty cycle: an array comprising 100 resonators could be sampled in a few 100 ms. This is not very relevant for today’s low-efficiency NEMS-MS systems, where the probability of multiple events within a duty cycle is very low. In the future, however, as system particle transfer efficiencies improve, this probability will certainly increase. To circumvent such problem, several arrays with frequency addressing could eventually be operated simultaneously in parallel with multiple-channel electronics.

We also demonstrate here how the frequency-addressing scheme can provide individual resonator information: the array becomes a sort of particle imager, each resonator acting as a pixel. With this technique, mass spectrometry imaging (MSI) at the single-particle level becomes possible. MSI generally relies on the analysis of localized desorption events sequentially in time and has already proven its great potential for clinical applications and cancer research^19^. More recent techniques perform multi-pixel images with one single shot and efforts in the field are pushing towards better resolution imagers^20^ as well as high-mass capability^21^. NEMS-MS imaging with frequency-addressed arrays has this potential. Ultimately, arrays of nanoresonators with µm-sized pixels covering large areas could be fabricated with CMOS co-integration^22^. Many frequency-addressed arrays could thus be operated simultaneously with an integrated electronics, like a CMOS imager. MS analysis of massive biological species could then be performed with NEMS-MS systems using gas-phase transfer techniques like electrospray ionization or surface acoustic wave nebulization, leading to limit of detection and efficiency similar to conventional MS. Moreover, the mass resolution of an array of NEMS does not theoretically depend on particle mass (in the same experimental conditions), improving the relative precision at higher mass. Beside biological research and biomedical applications, NEMS-MS imaging could be of great interest for the characterization of ionization sources efficiency, as well as the characterization of sampler performance in aerosol science.

## Methods

### Array fabrication

The NEMS arrays employed in this work are fabricated from CMOS-compatible materials and VLSI processes. They are fabricated from a 200-mm SOI wafer with 160-nm-thick silicon layer. The top silicon layer is implanted with boron ions (p-type, ~10^19^cm^−3^) resulting in silicon resistivity of a few mΩ·cm. A hybrid e-beam/DUV lithography technique is used and the top silicon layer is subsequently etched by anisotropic reactive ion etching (RIE). A silicon oxide layer is deposited and patterned to open the contact area between silicon and metal. A first AlSi layer is deposited and patterned to define metal leads. A second silicon oxide layer is deposited, planarized by chemical mechanical polishing and patterned to open the via holes for electrical contact between the first and second metal levels. The second AlSi layer is then deposited and patterned to define the top level electrical leads and wire-bonding pads. Finally, the devices are released by vapor HF isotropic etching.

### Mass and position calculation

When a particle of mass ∆*m* lands on a resonator at the position *x*, a frequency shift ∆*f*_*n*_ of the mode *n* occurs:

2 $$\Delta m = 2M\frac{{\Delta f_n}}{{f_n}}\frac{{\alpha _n}}{{\varphi _n\left( x \right)^2}}$$

with *f*_*n*_ and $\frac{{\varphi _n\left( x \right)^2}}{{\alpha _n}}$ being respectively the nominal frequency and the position dependent normalized mode shape of the mode *n*. Using the first two modes of the NEMS, we obtain:

3 $$\frac{{\varphi _1\left( x \right)^2}}{{\varphi _2\left( x \right)^2}} = \frac{{\alpha _1\frac{{\Delta f_1}}{{f_1}}}}{{\alpha _2\frac{{\Delta f_2}}{{f_2}}}}$$

The function$g\left( x \right) = \frac{{\varphi _1\left( x \right)^2}}{{\varphi _2\left( x \right)^2}}$ is invertible only on half of the beam *x* ∈ [0; 0.5[ or *x* ∈ ]0.5; 1], but as a doubly clamped beam as used here is symmetric, we can solve the position in only one of those two halves:

4 $$x = g^{ - 1}\left( {\frac{{\alpha _1\frac{{\Delta f_1}}{{f_1}}}}{{\alpha _2\frac{{\Delta f_2}}{{f_2}}}}} \right)\quad x \in \left[ {0;0.5} \right[$$

Then the mass of the landed particle can be calculated:

5 $$\Delta m = 2M\frac{{\Delta f_1}}{{f_1}}\frac{{\alpha _1}}{{\varphi _1\left( x \right)^2}} = 2M\frac{{\Delta f_2}}{{f_2}}\frac{{\alpha _2}}{{\varphi _2\left( x \right)^2}}$$

### Frequency stability measurements

In Fig. 2c, frequency stabilities are compared for three different cases. In each case, bias and drive voltages are increased until the frequency stability stopped improving (meaning the limiting source of noise is intrinsic to the resonator itself). This “limit” frequency stability is the one plotted for each case. It is obtained with 0.3 to 0.4 V drive voltage for both single and array resonators, 1.25 V bias voltage for a single resonator and 3.5 V bias voltage for the array.

Importantly, it should be noted that the standard Allan deviation cannot be used in the case of frequency addressing as there is dead time in between each frequency data point for a given resonator in the array^23^. In order to compare the frequency stabilities in the three cases shown, we measure frequency samples using the same integration time *τ*_PLL_ and dead time (*N* − 1)*τ*_PLL_ for the three cases. The measurement time for each sample is then *Nτ*_PLL_. We concatenate these samples (i.e., simply suppressed the dead times) and use the Allan deviation expression to obtain the plots: this estimator is useful for comparison purposes, but should not be mistaken with rigorous Allan deviation measurements. This estimator has also been chosen for Supplementary Figure 4b. Supplementary Figure 4a, however, uses the standard Allan deviation procedure.

### Mass sensitivity calibration

Calibration of the NEMS sensitivity is essential for a correct comparison between TOF-MS and NEMS-MS^6^. The NEMS frequency response to uniform mass deposition is compared to mass deposition rates provided by a QCM. This procedure is all the more relevant with arrays as all resonators vary in length and consequently in mass sensitivity. These sensitivities need to be measured for each individual resonator in the array. This can be performed simultaneously with the frequency-addressing technique: the NEMS array is introduced in particle deposition chamber of the setup. The cluster source is adjusted to produce a steady flow of metallic nanoparticles, delivered either to the NEMS array or the QCM. Several acquisitions with increasing deposition rates are performed with both the NEMS array and the QCM. Measurements of both mode 1 and mode 2 using the frequency-addressing scheme provides the frequency traces of every resonator for both modes. The different mass sensitivities are obtained by comparing the NEMS frequency shift rates to the particle deposition rate given by the QCM, as shown in Supplementary Figure 5, and using the known length and width of each resonator in the array.

It should be noted that all device characterizations (frequency response, frequency stability, mass calibration) are performed after the device has reached thermal equilibrium. Indeed, a current flows in the nanogauges due to bias voltage, which in turns induces a rise in temperature due to Joule heating. We estimate this rise to be limited below 10 K.

### Data availability

The data that support the findings in this study are available from the corresponding author upon reasonable request

## Electronic supplementary material

Supplementary Information
