# Supplementary material for: Single-particle mass spectrometry with arrays of frequency-addressed nanomechanical resonators
Source: Nat Commun. 2018 Aug 16;9:3283. doi: 10.1038/s41467-018-05783-4 (PMC6095856; doi:10.1038/s41467-018-05783-4)
Supplement: Supplementary file 1 — Supplementary Information [file 41467_2018_5783_MOESM1_ESM.pdf]

Single-particle Mass Spectrometry with arrays of frequency-addressed nanomechanical resonators

Sage et al

SUPPLEMENTARY INFORMATION

Supplementary Figures

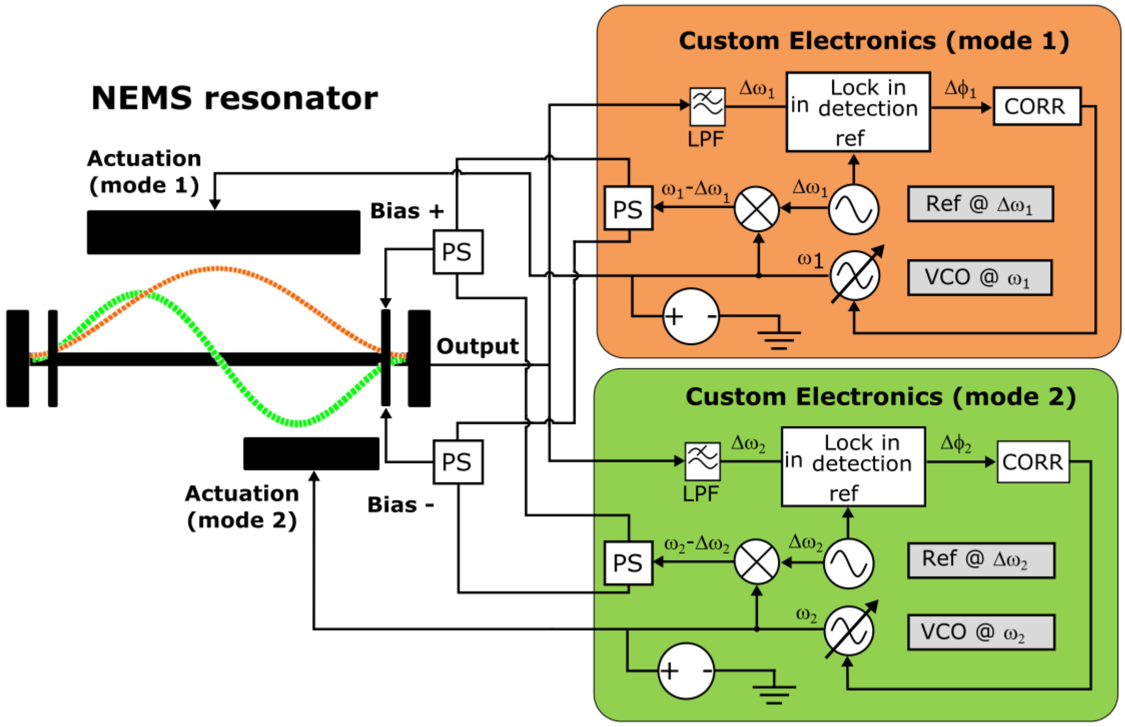

Supplementary Figure 1: Diagram of the NEMS array and its associated custom electronics to simultaneously track mode 1 and mode 2 resonances with PLLs. For the sake of readability, only one resonator is shown on the figure, but the layout is identical for an array. PS: Power Splitter (or combiner) ; LPF: Low Pass Filter. For each mode the beam is driven at ω (some tens of MHz) and the bias frequency is at $\omega - \Delta\omega$ to downmix the output at $\Delta\omega$ (some tens of kHz) and circumvent signal attenuation caused by parasitic impedances. Typical drive and bias voltages for our arrays are a few 100mV and a few V respectively. In Closed Loop operation, the output signal phase difference is shaped by a corrector (CORR) that feeds a Voltage Controlled Oscillator (VCO) driving the NEMS resonator at its resonance frequency. The corrector is a robust H∞ controller whose coefficients are calculated using a loop shaping method[1].

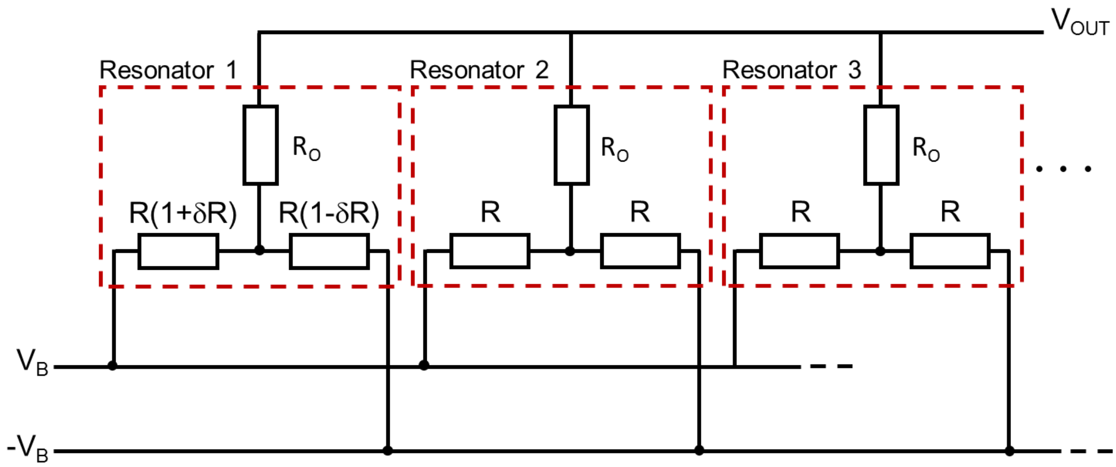

Supplementary Figure 2: Equivalent electrical circuit of a resonator array. Each resonator is modelled as 2 gauge resistances (R) and an output resistance (R_O). Only one resonator is vibrating at a given time, in this case Resonator 1: for that resonator, the gauges' resistance is modelled as $R(1 \pm \delta R)$, where δR is the motional component of the resistance.

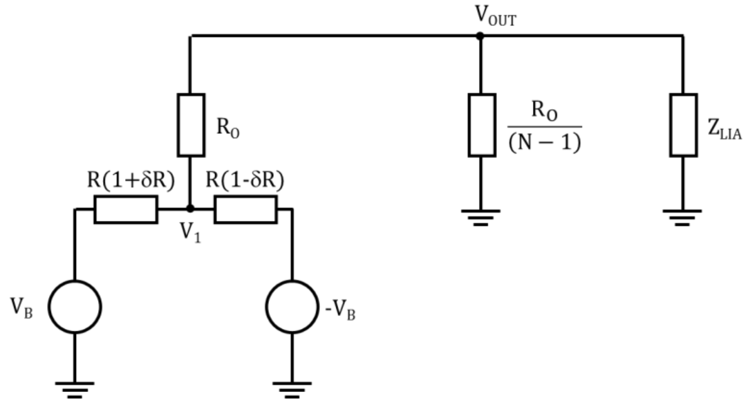

Supplementary Figure 3: Equivalent electrical circuit of the measurement of an array in frequential addressing mode. The output measured signal V_{OUT} is measured using a lock-in amplifier, with impedance Z_{LIA}.

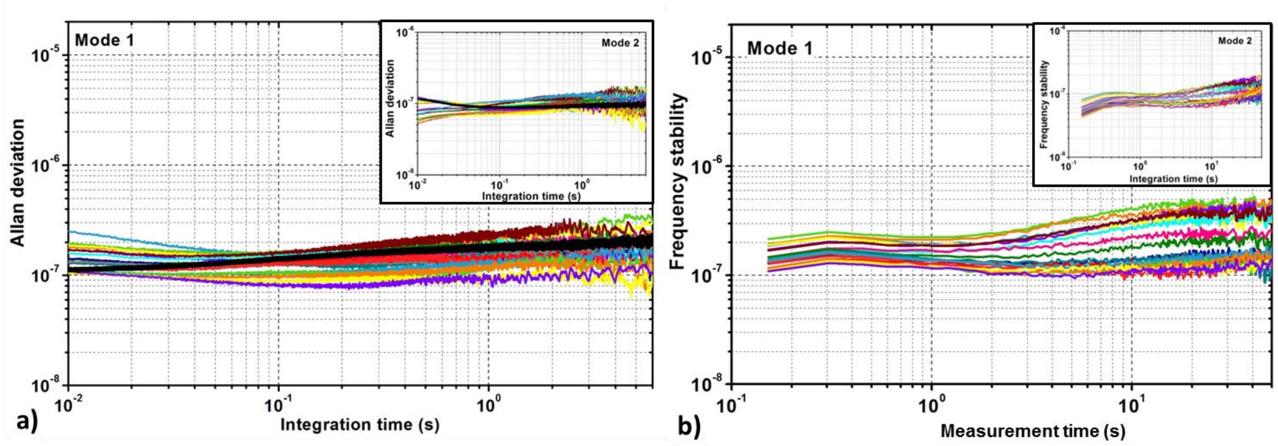

Supplementary Figure 4: Frequency stability characterization. a) Solid black line: Allan deviation of a single resonator (not in array) at liquid nitrogen temperature with dimensions equal to those of the longest resonator in the array. Solid color lines: Allan Deviations of all 19 resonators in an array at liquid nitrogen temperature, measured individually (without frequency addressing). Mode 1 in main figure, mode 2 in inset. b) Frequency stability of all 19 resonators in an array at liquid nitrogen temperature with the frequency addressing scheme (see description of estimator above). The difference in estimators explains the difference in time scale. Mode 1 in main figure, mode 2 in inset.

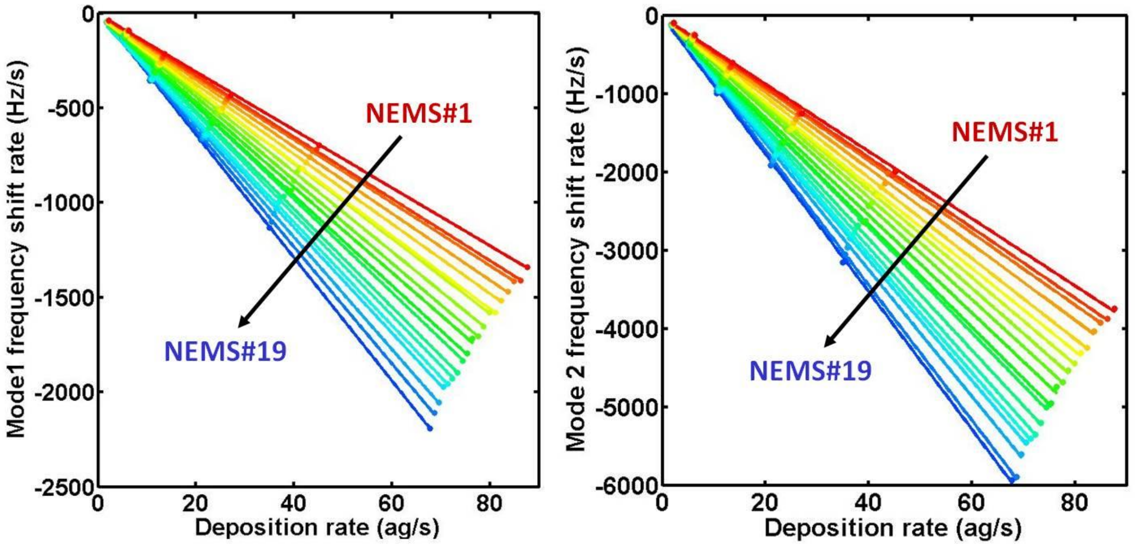

Supplementary Figure 5: Mode 1 and mode 2 linear fits of frequency shift rates with an array of 19 NEMS with respect to the deposition rate on their surface measured with a QCM. Red curve corresponds to NEMS#1 and the color progressively turn to blue for smaller devices until dark blue for NEMS#19. We deposited a maximum mass of ~ 4 fg << ~ 1 pg (total resonator mass) to remain in the linear regime. This comparison yields the product $\boldsymbol{\rho.t}$ with t the thickness of the resonator and $\boldsymbol{\rho}$ its density. Mass sensitivity and effective mass for each mode is finally calculated from this product, width and length of each resonator.

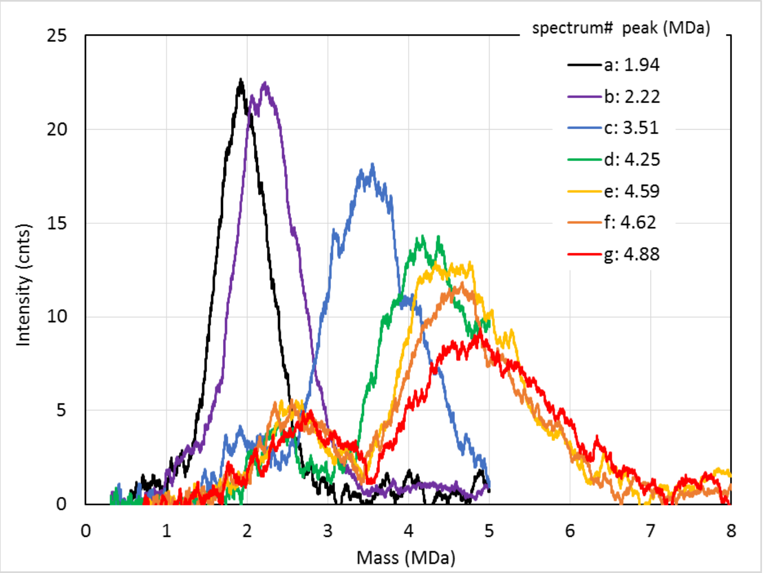

Supplementary Figure 6: TOF spectra obtained with tantalum clusters of increasing size and mass. The plot shows that both intensity and width of peaks degrade very quickly beyond 2MDa. There are two reasons for this degradation: first heavy ions are less accelerated and the ion detector of the TOF Mass Spectrometer becomes less efficient[2]. Secondly, the cluster source might produce a lower number of aggregates. Because of this degradation, we limited our mass range of operation below 3MDa.

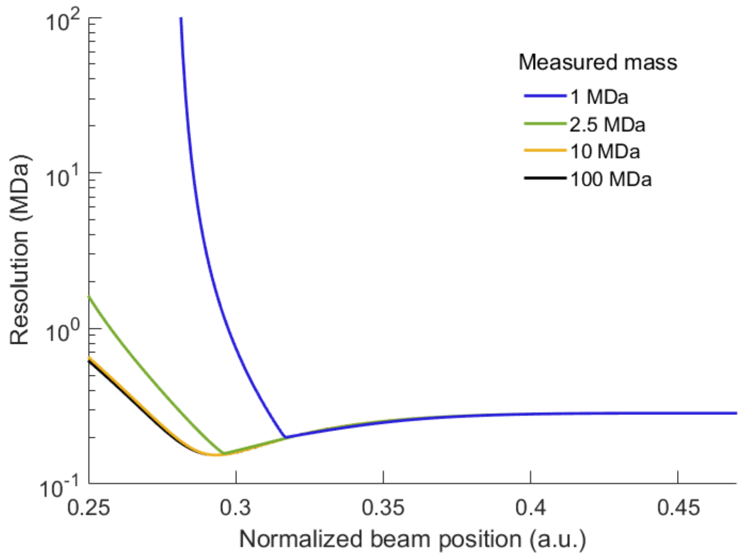

Supplementary Figure 7: Mass resolution for increasing masses. These numerical calculations present the mass uncertainty obtained for particles of different masses, obtained from the measured frequency stability of a given NEMS in the array. This mass uncertainty depends on the landing position on the beam (normalized, with 0 and 1 being the clamped ends and 0.5 the center). Only a fixed (shown here) portion of the beam is considered in all the analysis in order to obtain a chosen mass resolution. This mass uncertainty increases when the particle lands close to the anchors, where the motion becomes small. How close and how much the resolution increases depends on the particle mass. But for high enough masses (>2.5MDa), the mass resolution does not depend on measured mass anymore. This shows that the resolving power of NEMS-MS increases for heavier particles, with proportionally narrower peaks.

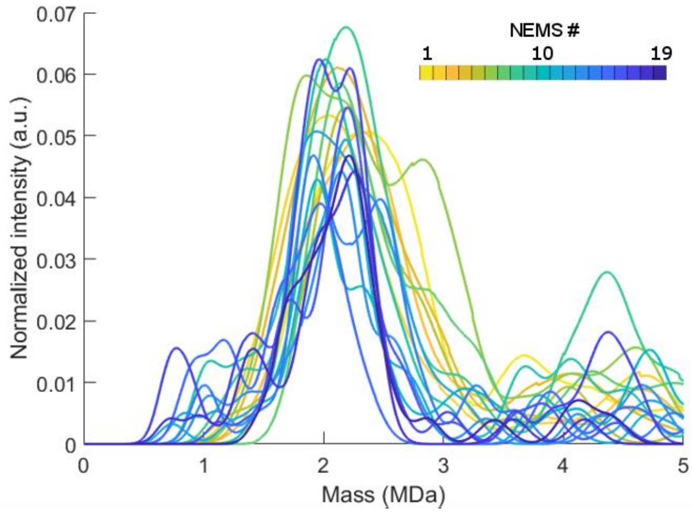

Supplementary Figure 8: Mass spectrum of each individual NEMS for the 7.4 nm tantalum clusters. Each spectrum obtained with every individual NEMS is represented here with a different color. Their intensities are normalized by the total number of events in the overall spectrum. This plot clearly shows the variability across the array and how this variability contributes to the broadness of the overall peak.

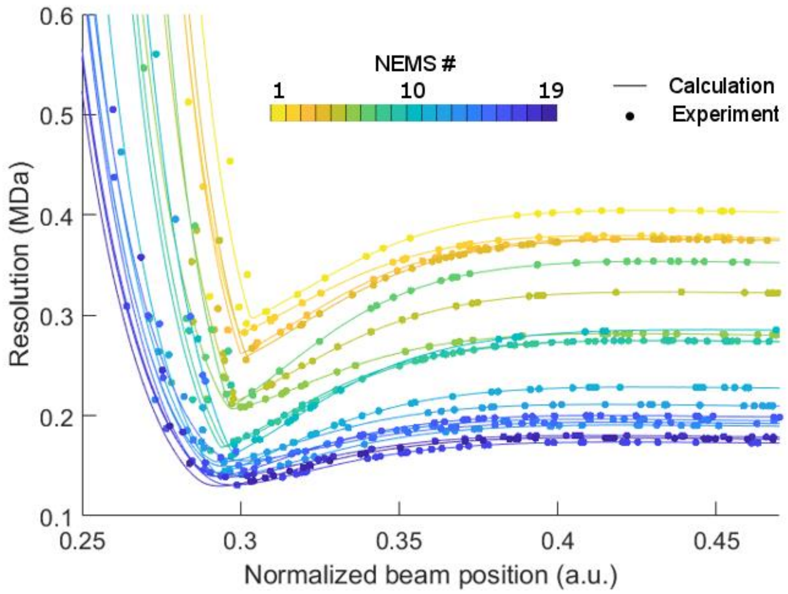

Supplementary Figure 9: Mass uncertainty of every detected particle for the 7.4 nm tantalum clusters. Each dot is the mass uncertainty of a detected particle obtained from the measured frequency stability of the resonator (both modes) on which the particle landed. The x axis is the position of the particle along the beam normalized by the length of the beam (0 is an anchored end, 0.5 is the middle of the beam). Each NEMS in the array is associated to a different color in the plot. The solid lines show the theoretical mass uncertainties calculated for particles of a fixed 2.15MDa mass. This plot shows that while the average mass resolution is about 250kDa across all events and all NEMS, *ie* very similar to an individual NEMS, there is a large spread in mass uncertainties. This mass uncertainty is proportional to both effective mass of the resonator and its frequency stability. While the relation between the former to resonator length is well established, the latter is less known. This explains why mass uncertainty in the plot is not a monotonic function of resonator length. In any case, this spread contributes to the broadness of the overall spectrum.

Supplementary Methods

Electrical signal with arrays

In a frequency addressed architecture, all N resonators are connected in parallel and the array can be modeled as shown in Supplementary Figure 2. At a given time, only one of the resonators is in motion, so the resistance of the nanogauges in all the others is balanced, resulting in a virtual ground between them. In this case, the equivalent circuit is as shown Supplementary Figure 3, very similar to that of a single resonator.

The load impedance at the output of the resonator is $Z_{out} = Z_{LIA} // \frac{R_0}{N-1} \approx \frac{R_0}{N-1}$, as $Z_{LIA} \approx 10k\,\Omega$ and $R_0 \approx 1k\,\Omega$ We can then use Millman's theorem to find the voltage V_1:

$$V_1 = -\frac{V_b \times \delta R \times (R_0 + Z_{out})}{R_0 + Z_{out} + \frac{R(1-\delta R^2)}{2}} \approx \frac{V_b \times \delta R \times R_0}{R_0 + \frac{R}{2}}$$

The approximation is valid when N is large, and $\frac{R_0}{N-1} \ll R_0$. The measurement voltage is then

$$V_{out} = V_1 \frac{Z_{out}}{R_0 + Z_{out}} = -\frac{\mathrm{V_b \times \delta R \times R_0}}{\mathrm{R_0 + \frac{R}{2}}} \frac{\frac{R_0}{N-1}}{\mathrm{R_0} + \frac{R_0}{N-1}} \approx -\frac{\mathrm{V_b \times \delta R}}{\mathrm{N-1}} \frac{\mathrm{R_0}}{\mathrm{R_0 + \frac{R}{2}}}$$

Supplementary References

1. Kharrat, C., Colinet, E. & Alina, V. H Loop shaping control for PLL-based mechanical resonance tracking in NEMS resonant mass sensors. *2008 IEEE Sensors, SENSORS 2008, Oct. 26, 2008 - Oct. 29, 2009* 1135–1138 (2008). doi:10.1109/ICSENS.2008.4716641
2. Westmacott, G., Frank, M., Labov, S. & Benner, W. Using a superconducting tunnel junction detector to measure the secondary electron emission efficiency for a microchannel plate detector bombarded by large molecular ions. *Rapid Commun. Mass Spectrom.* **14,** 1854–61 (2000).
